# miR‐372 and miR‐373 enhance the stemness of colorectal cancer cells by repressing differentiation signaling pathways

**DOI:** 10.1002/1878-0261.12376

**Published:** 2018-09-24

**Authors:** Lu‐Qin Wang, Peng Yu, Bin Li, Yan‐Hua Guo, Zi‐Rui Liang, Ling‐Ling Zheng, Jian‐Hua Yang, Hui Xu, Shun Liu, Li‐Si Zheng, Hui Zhou, Liang‐Hu Qu

**Affiliations:** ^1^ Key Laboratory of Gene Engineering of the Ministry of Education State Key Laboratory of Biocontrol School of Life Sciences Sun Yat‐sen University Guangzhou China

**Keywords:** cancer stem cell, colorectal cancer, miR‐372/373, NFκB, SPOP, VDR

## Abstract

miR‐372/373, a cluster of stem cell‐specific microRNAs transactivated by the Wnt pathway, has been reported to be dysregulated in various cancers, particularly colorectal cancer (CRC); however, the unique role of these microRNAs in cancer remains to be discovered. In the present study, we characterized the upregulation in expression of miR‐372/373 in CRC tissues from The Cancer Genome Atlas data, and then showed that overexpression of miR‐372/373 enhanced the stemness of CRC cells by enriching the CD26/CD24‐positive cell population and promoting self‐renewal, chemotherapy resistance and the invasive potential of CRC cells. To clarify the mechanism underlying microRNA‐induced stemness, we profiled 45 cell signaling pathways in CRC cells overexpressing miR‐372/373 and found that stemness‐related pathways, such as Nanog and Hedgehog, were upregulated. Instead, differentiation‐related pathways, such as NFκB, MAPK/Erk and VDR, were markedly repressed by miR‐372/373. Numerous new targets of miR‐372/373 were identified, including SPOP, VDR and SETD7, all of which are factors important for cell differentiation. Furthermore, in contrast to the increase in miR‐372/373 expression in CRC tissues, the expression levels of SPOP and VDR mRNA were significantly downregulated in these tissues, indicative of the poor differentiation status of CRC. Taken together, our findings suggest that miR‐372/373 enhance CRC cell stemness by repressing the expression of differentiation genes. These results provide new insights for understanding the function and mechanisms of stem cell‐specific microRNAs in the development of metastasis and drug resistance in CRC.

**Abbreviations**
CRC: colorectal cancer
CSC: cancer stem cell
DMEM: Dulbecco's modified Eagle's medium
ESCs: embryonic stem cells
FACS: fluorescence activated cell sorting
GICs: glioma initiation cells
miRNA: microRNA
sRNA: small RNA
TCGA: The Cancer Genome Atlas

## 1 Introduction

MicroRNAs (miRNAs) are a class of small non‐coding RNAs of 20–25 nucleotides in length that bind to the 3′ UTR of specific target mRNAs to regulate global gene expression by suppressing protein translation (Bartel, 2004). Previous studies showed that most miRNAs are limited to specific stages in embryonic development (Landgraf *et al*., 2007; Wienholds *et al*., 2005) and are often deregulated during tumorigenesis (Calin *et al*., 2004; Volinia *et al*., 2006), and also they regulate developmental events throughout embryogenesis (Ivey and Srivastava, 2010) and tumor development in various cancers (Garzon *et al*., 2006). miRNA‐372 and miRNA‐373 (miR‐372/373) were originally found to be expressed specifically in human embryonic stem cells (ESCs) (Suh *et al*., 2004). It is reported that enforced expression of miRNA‐372 promoted reprogramming of human fibroblasts to induce pluripotent stem cells by targeting transforming growth factor β signaling (Subramanyam *et al*., 2011), indicating that miRNA‐372 participate in the process of pluripotency induction.

Colorectal cancer (CRC) is one of the most common cancers worldwide. It is reported that approximately 90% of CRC have an activation mutation of Wnt/β‐catenin pathway (Schneikert and Behrens, 2007). Wnt signaling has been reported to maintain CRC stem cell‐like properties (Vermeulen *et al*., 2010), which are proposed to be a major cause of treatment resistance and recurrence of cancer (Creighton *et al*., 2009). As we previously found that Wnt/β‐catenin transactivated miR‐372/373 in CRC and identified a positive feedback loop (Zhou *et al*., 2012), it is highly likely that upregulation of miR‐372/373 contributes to cancer stem cell (CSC)‐like properties in CRC. However, currently, the functions of miR‐372/373 in CRC are still debatable. There are some reports showing that miR‐372/373 are upregulated in CRC, promote metastasis and predict poor prognosis (Eyking *et al*., 2016; Loayza‐Puch *et al*., 2010; Yamashita *et al*., 2012). There are also reports indicating that miR‐373 is downregulated and inhibits tumor growth in CRC (Tanaka *et al*., 2011). The expression of miR‐372/373 in CRC tissue samples has not yet been evaluated on a large scale, and the mechanism underlying CRC development regulated by miR‐372/373 has not been systematically decoded.

In the present study, we first analyzed the expression of miR‐372/373 in CRC samples from The Cancer Genome Atlas (TCGA) database and noted the relationship between miR‐372/373 and stem cell markers in CRC cell lines. We both stably overexpressed and knocked down miR‐372 and miR‐373 in CRC cell lines and demonstrated stemness change in both the molecular markers and phenotypes of CRC cells. When deciphering the molecular mechanism underlying miRNA‐induced stemness, we determined the 45 cell signaling pathways in CRC cells overexpressing miR‐372/373 and identified multiple new target genes of miR‐372/373 in CRC cells. These results reveal that miR‐372/373 promotes CRC stem cell traits via repression differentiation signaling.

## 2 Materials and methods

### 2.1 Cell culture and transfection

HCT116 and 293FT cells were maintained in Dulbecco's modified Eagle's medium (DMEM) supplemented with 10% fetal bovine serum. RKO cells were maintained in MEM with 10% fetal bovine serum. Caco‐2 cells were maintained in MEM medium with 20% fetal bovine serum. HCT116, RKO and Caco‐2 cells were purchased from the Chinese Science Academy (Beijing,China) and 293FT cells were purchased from Invitrogen (Carlsbad, CA, USA). All cells were cultured at 37 °C with 5% CO_2_.

All cell transfections were performed using Lipofectamine 2000 transfection reagent (Invitrogen) in accordance with the manufacturer's instruction. RNA (Ribo, Guangzhou, China) transfections were performed at a final concentration of 50 nm.

### 2.2 Generation of stable cell lines

Establishment of the HCT116 pISNCG‐miR‐372/373 and RKO pISNCG‐miR‐372/373 stable cell lines was performed as described previously (Xu *et al*., 2010). Briefly, lentiviruses were generated by co‐transfection 3 μg of transfer vector and 9 μg of packaging mix in 293FT cells. Supernatants were collected 48 h after transfection, filtered through a 0.45 μm membrane and used to infect cells in the presence of polybrene (6 μg·mL^−1^). Geneticin (1000 μg·mL^−1^) was used to select positive cells.

Establishment of the Caco‐2 TuD‐miR‐372/373 stable cells was performed using pGreen‐Puro lentiviral vector (System Biosciences, Palo Alto, CA, USA). Lentiviruses were generated by co‐transfection using 12 μg of transfer vector and 12 μg of packaging mix in 293FT cells. Puromycin (1 μg·mL^−1^) was used to select positive cells.

### 2.3 miRNA sequencing

Total RNA was extracted using TRIzol reagent (Invitrogen). The small RNA (sRNA) was isolated by separating 10 μg of total RNA on denaturing polyacrylamide gel electrophoresis and excising the portion of the gel corresponding to 18–30 nucleotides based on oligonucleotide markers. Adapters (5′ and 3′) were ligated to the sRNA population. Modified sRNAs were reverse transcribed and PCR‐amplified with adapter‐specific primers. The amplified cDNAs were purified by urea‐polyacrylamide gel electrophoresis for sequencing with a genome analyzer (Illumina, Inc., San Diego, CA, USA).

### 2.4 Flow cytometry assay

Cells were digested by trypsin and 1 × 10^6^ cells were suspended in 100 μL of PBS for each assay. For CD133 detection, the cell suspension was stained with a CD133 fluorescence‐labeled antibody (Miltenyi Biotec, Bergisch Gladbach, Germany) at 4 °C for 10 min. For CD44, CD24 and CD26 detection, the cell suspensions were stained with CD44, CD24 and CD26 fluorescence‐labeled antibodies (eBioscience, San Diego, CA, USA), respectively, at 37 °C for 30 min. The negative control included cells incubated with isotype control antibodies for each color. Fluorescence signals were analyzed using fluorescence activated cell sorting (FACS) on a BD FACSCalibur flow cytometer (BD Biosciences, San Jose, CA, USA).

### 2.5 Sphere formation assay

Cells (3 × 10^3^) were seeded in six‐well ultra‐low attachment surface plates (Corning, New York, NY, USA) and cultured in DMEM/F12 serum‐free medium (Gibco, Life Technologies, Scoresby, VIC, Australia) supplemented with B27 (Invitrogen), 20 ng·mL^−1^ of EGF (Invitrogen) and 20 ng·mL^−1^ of bFGF (Invitrogen) for 7 days. The spheres were photographed and counted under a Zeiss Axio Observer.Z1 (Carl Zeiss, Oberkochen, Germany) at a magnification of 20×.

### 2.6 5‐Fluorouracil treatment

HCT116 stable cells were collected and 3 × 10^4^ cells were seeded in 24‐well plates and incubated at 37 °C in a humidified CO_2_ incubator. After 24 h, the medium was aspirated and replaced with growth media containing 5, 10, 15, 20 or 25 μm 5‐fluorouracil (Sigma‐Aldrich, St Louis, MO, USA). After 72 h, the viable cells were collected and seeded in 96‐well plates and examined using a Cell Counting Kit‐8 (Dojindo, Shanghai, China).

### 2.7 Migration and invasion assay

For migration and invasion assays, the cells were suspended in 200 μL of medium without fetal bovine serum and were then seeded into the upper chamber of Transwell inserts (8 μm pore size; Corning) with or without a Matrigel (R&D Systems, Minneapolis, MN, USA) coating. The lower chamber of the Transwell was filled with 750 μL of medium supplemented with 10% fetal bovine serum, which functions as a chemoattractant. After 24 h of incubation at 37 °C, cells that migrated or invaded the lower surface of the insert membrane were fixed in methanol and stained with 0.1% crystal violet. The cells that migrated or invaded were photographed under a Zeiss Axio Observer.Z1 (Carl Zeiss) at a magnification of 20×. Numbers of cells were analyzed by image pro‐plus (Media Cybernetics, Inc., Bethesda, MD, USA).

### 2.8 Animal study

The 4–6‐week‐old female BALB/c nude mice were purchased from Guangdong Medical Laboratory Animal Center (Guangzhou, China). The experiments were performed in accordance with the Guidelines for the Care and Use of Laboratory Animals (NIH publication No. 80‐23, revised 1996) and according to the ethical principles for experiments on animals of Institutional Animal Care and Use Committee of Sun Yat‐sen University. For each group, five mice were used. For each mouse, 2 × 10^6^ HCT116 cells suspended in 200 μL of 1 × PBS were injected subcutaneously into the dorsal flank. Tumor growth was confirmed and recorded by two investigators. The mice were euthanized 20 days after injection. Tumor tissue protein was extracted by TRIzol reagent (Invitrogen).

### 2.9 Quantitative RT‐PCR

Total RNA was extracted by TRIzol reagent (Invitrogen). Quantitative RT‐PCR assays were per‐formed using SYBR PrimeScript™RT‐PCR kit (Takara Bio Inc., Otsu, Japan) as described previously (Xu *et al*., 2010). For reverse transcription reactions, stem loop reverse transcript primers were used for miRNAs and oligo dT mixed with random primers was used for mRNAs. The primers used for real‐time PCR are listed in Table S1. The specificities of primer were analyzed both using *in silico* primer‐blast and melting curve detection.

### 2.10 Western blotting

Protein samples were extracted with TRIzol reagent (Invitrogen) and were dissolved in an amphoteric electrolyte. Western blot assays were performed as described previously (Huang *et al*., 2012). Nitrocellulose membranes were blocked using 5% Blotting Grade Blocker Non‐Fat Dry Milk (Bio‐Rad, Hercules, CA, USA) and were then incubated with primary antibodies at 4 °C overnight. For primary antibodies, anti‐CD26 was purchased from R&D Systems (Minneapolis, MN, USA); anti‐CD44, anti‐p65, anti‐p‐p65, anti‐SETD7 anti‐VDR and anti‐GAPDH were purchased from Cell Signaling Technology (CST, Danvers, MA, USA); and anti‐SPOP was purchased from Proteintech (Wuhan, China). The blots were then incubated with horseradish peroxidase‐conjugated secondary antibody (CST) at room temperature for 1 h. Bands were analyzed by gel‐pro analyzer software (Media Cybernetics).

### 2.11 Vector construction

For the stable miRNA expressing vector, an approximately 300 bp DNA fragment containing pre‐miR‐372 or pre‐miR‐373 was amplified from SW480 genomic DNA and cloned into the pISNCG vector. For miRNA transient overexpression vectors, the 300 bp DNA fragment containing the entire pre‐miR‐372 or pre‐miR‐373 sequence was cloned into the pcDNA6.2 vector (K4936‐00; Invitrogen). For the stable miR‐TuD‐expressing vector, the 146 nt TuD stem loop sequence, which could sequester miR‐372/373, was synthesized and then cloned into the pGreen‐Puro vector (System Bioscience).

For target validation, both strands of the 59 nucleotide 3′ UTR sequence containing the miRNA binding region of each target gene were synthesized, directly annealed and then cloned into the psiCheck2 vector (Promega, Madison, WI, USA).

For gene overexpression vectors, the RNA was reverse transcribed and fragments containing the VDR, SPOP and SETD7 were amplified and cloned into the pcDNA3.1 vector (V790‐20; Invitrogen).

Cignal 45‐pathway reporter arrays that measure the activity of the 45 pathway were purchased from Qiagen (Hilden, Germany).

Primers for vector construction are listed in Table S2.

### 2.12 Luciferase reporter assay

For pathway activity assays, 3.5 × 10^4^ HCT116 cells were plated in 48‐well plates and transfected with 50 ng of pGL4.11‐enhancer reporter vectors and 2.5 ng of pRL‐TK *Renilla* control vector. For target 3′ UTR luciferase assays, HCT116 cells were plated in 48‐well plates and transfected with 100 ng of pcDNA6.2‐miR‐372/miR‐373 and 100 ng of psiCheck2 target 3′UTR vector. After 48 h, the luciferase assay was performed using a Dual‐Luciferase Reporter Assay System (Promega) on a GloMax 96 Microplate Luminometer (Promega).

### 2.13 Statistical analysis

The data were presented as the mean ± SEM of three separate experiments, unless otherwise stated. If the data followed Gaussian distributions, a Student's *t*‐test was conducted. If the data did not follow Gaussian distributions, the Wilcoxon rank‐sum test was used. The RNA‐sequencing data and the corresponding clinical information were downloaded from TCGA. The clinical correlation was analyzed by the chi‐squared test. *P* < 0.05 was considered statistically significant.

## 3 Results

### 3.1 miR‐372/373 are upregulated in CRC and involved in CSC properties

**Figure 1:**
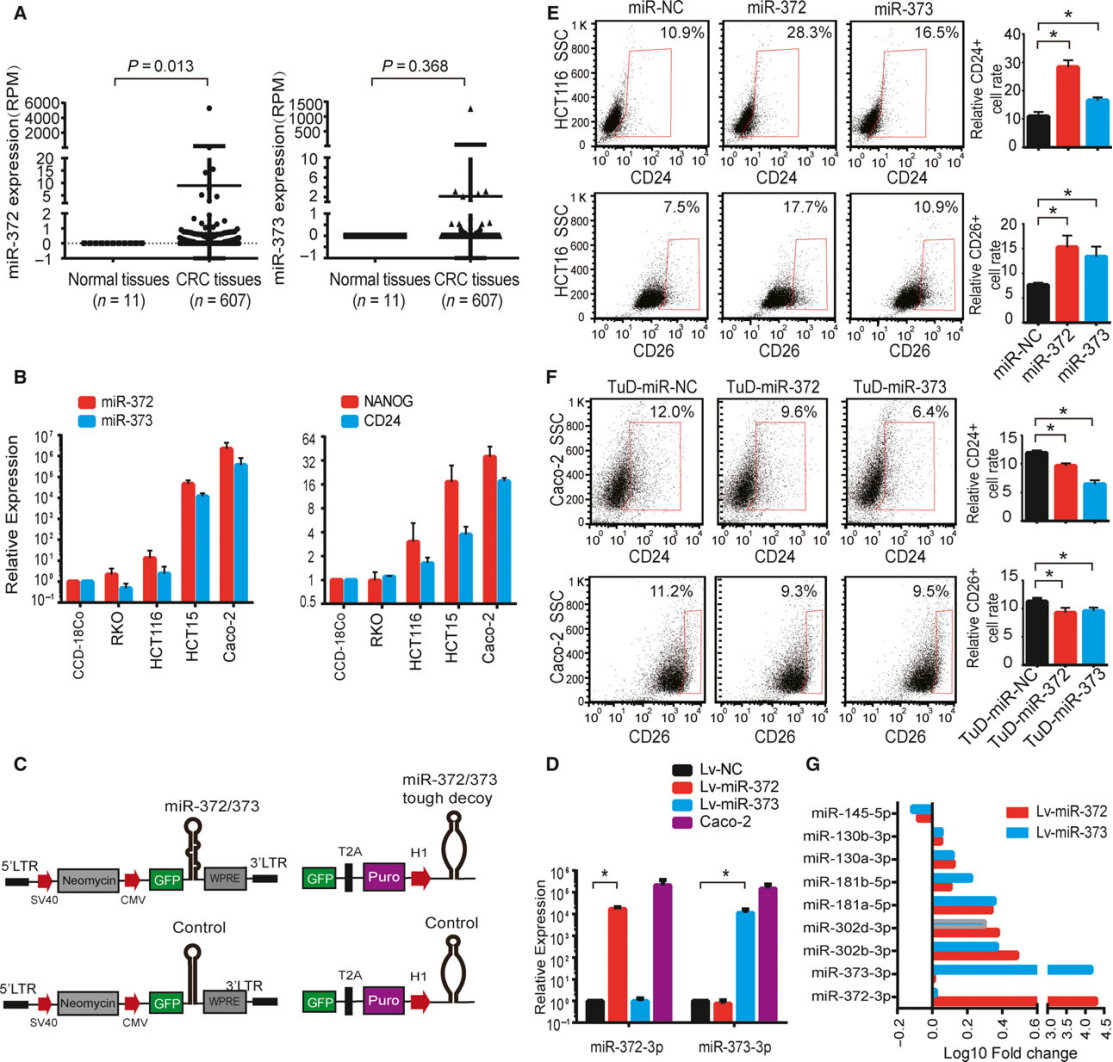
miR‐372/373 expand the CRC stem cell population. (A) The expression of miRNA‐372/373 in CRC and healthy colon samples from TCGA database. (B) Quantitative RT‐PCR showing relative expression of the miR‐372/373 and mRNA of Nanog and CD24 in different cell lines. The data were log‐transformed after being normalized to the U6 internal control and the normal colon cell control CCD‐18Co. (C) The scheme shows the lentivirus system for generating stable miR‐372/373‐expressing and miR‐372/373‐touch decoy (TuD)‐expressing cell lines. (D) miRNA‐372 and miR‐373 levels in HCT116‐Lv‐miR‐372 and HCT116‐Lv‐miR‐373 compared to HCT116‐miR‐NC stable cells and Caco‐2 cells measured by quantitative RT‐PCR. (E) Relative CD24+ and CD26+ cell populations in the indicated HCT116 stable cells determined by FACS. (F) Relative CD24+ and CD26+ cell populations in the indicated Caco‐2 miR‐372/373‐TuD stable cells determined by FACS. (G) miRNA profiling showing selected deregulated miRNAs in HCT116‐miR‐372/373 stable cells. Error bars represent the SEM (*n* = 3). *P <* *0.05 by Student's *t*‐test.

To study the expression pattern of miR‐372/373 in CRC, we characterized the expression of miR‐372/373 in 607 CRC tissue samples and 11 adjacent normal colon tissues available from TCGA. We found that miR‐372/373 were not expressed in normal adjacent colon tissues, although they were highly expressed in lots of CRC tissues (Fig. 1A). We further examined the expression of the miR‐372/373 in CRC cell lines and found that miR‐372/373 were high in CRC cell lines Caco‐2 and HCT15 cells; low in CRC cell lines RKO and HCT116; and almost not expressed in normal human colon CCD‐18Co cells (Fig. 1B). These data indicate that expression of miR‐372/373 could be associated with a characteristic of CRC. To explore the possible involvement of miR‐372/373 in CSC properties, we examined the expression of the CSC markers Nanog and CD24 genes in CRC cell lines. As shown in Figs 1B and S1A, the expression patterns of miR‐372/373 were significantly correlated with Nanog and CD24 in these cell lines, suggesting that the miR‐372/373 are related to CSC features.

To confirm the involvement of miR‐372/373 in CSC properties, a lentivirus system stably overexpressing miR‐372 and miR‐373 or their decoy RNAs in CRC cell lines was established (Fig. 1C). We first overexpressed miR‐372/373 in HCT116 cells because these cells express low endogenous miR‐372/373. Significant upregulation of miR‐372/373 in these stably overexpressed cells was detected and their expression levels in HCT116 overexpressed cells are within the scope of a patient derived Caco‐2 cell line (Fig. 1D). We assessed the features of the cell population using FACS analysis. CD44, CD24, CD133 and CD26 were previously identified as CSC surface markers and as indicators of higher tumorigenic capability or with an invasive potential for CRC (King *et al*., 2012; O'Brien *et al*., 2007; Pang *et al*., 2010; Yeung *et al*., 2010). FACS analysis demonstrated that the percentage of CD24+, CD26+ (Fig. 1E), CD44+ and CD133+ (Fig. S2A) cells was significantly increased in miR‐372/373‐expressing HCT116 cells. RKO cells that also express low endogenous miR‐372/373 levels were analyzed. FACS analysis showed that RKO cells stably overexpressing miR‐372/373 had increased CSC surface markers (Figs S1B and S2B). By contrast to in HCT116 cells, miR‐372/373 are among the most abundant endogenous miRNAs in Caco‐2 cells. We therefore established new Caco‐2 cells lines stably expressing touch decoy RNAs (Haraguchi *et al*., 2009) against miRNA‐372 and miR‐373 (denoted TuD‐miR‐372/373) to achieve long‐term suppression of miR‐372/373 activity (Figs 1C and S1B,C). FACS analysis revealed that the competitive inhibition of miR‐372/373 reduced the CD24+ and CD26+ cell populations in Caco‐2 cells (Fig. 1F). These results indicate that miR‐372/373 are positively correlated with the CSC population in various CRC cells.

Moreover, to evaluate the impact of an ectopic miRNA with respect to CRC cells, we analyzed the miRNA profiles by deep sequencing in HCT116 cells overexpressing miR‐372/373. In addition to the significant upregulation of miR‐372/373, a large amount of miRNA was changed compared to the control. Particularly, a series of stem cell‐specific miRNAs, such as miR‐302 (Bourguignon *et al*., 2012; Lin *et al*., 2011; Subramanyam *et al*., 2011), miR‐130 (Ma *et al*., 2010; Pfaff *et al*., 2011) and miR‐181 (Ji *et al*., 2009, 2011; Judson *et al*., 2013; Wang *et al*., 2011), were upregulated, and stemness‐depressed miRNAs, such as miR‐145 (Jia *et al*., 2012; Ren *et al*., 2013; Xu *et al*., 2009), were downregulated, in miR‐372/373‐expressing cells (Fig. 1G). These results indicated that the overexpression of miR‐372/373 enables stem‐related miRNAs to change and synergistically promote stem‐like properties with respect to HCT116 cells.

### 3.2 Overexpression of miR‐372/373 promoted CSC phenotype of CRC cells *in vitro* and *in vivo*

**Figure 2:**
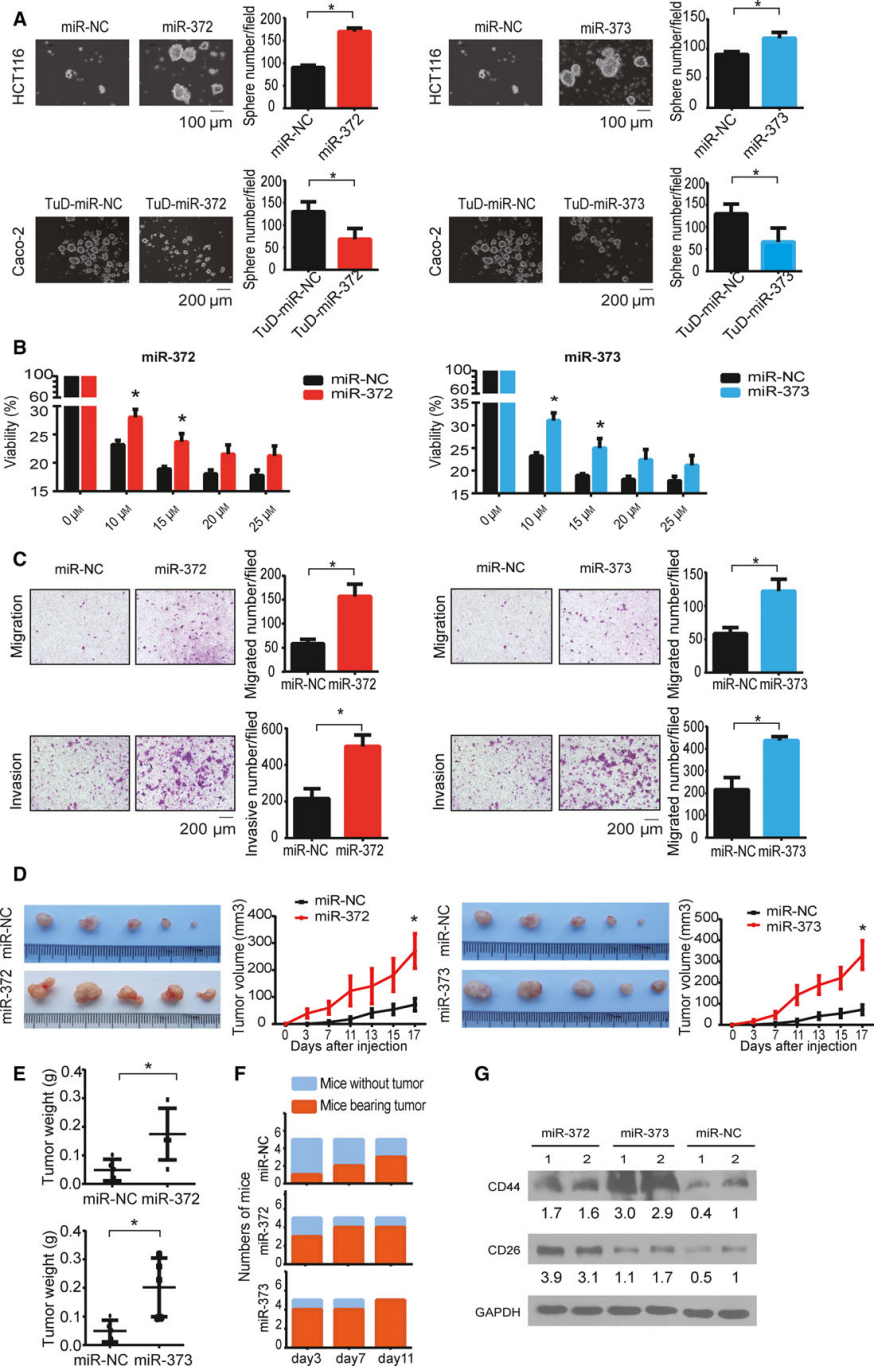
miR‐372/373 induce CRC stem cell phenotypes. (A) Representative images of spheres formed by the indicated HCT116 (scale bar = 100 μm) and Caco‐2 stable cells (scale bar = 200 μm) after 7 days of culture (left) and statistical analysis of the sphere formation rate (right). (B) CCK‐8 analysis of cell viability of HCT116‐miR‐372/373 cells treated with different doses of 5‐Fu for 72 h compared to the control. (C) Ectopic expression of miR‐372 and miR‐373 promoted migration and invasion of HCT116 cells. Left: representative images of migration and invasion assays with the indicated HCT116 stable cells. Scale bar = 200 μm. Right: mean number of cells per visual field was determined in three randomly selected visual fields per chamber, and the experiments were performed in triplicate. (D) Images and tumor growth curve of subcutaneous tumors derived from HCT116 cells. (E) Subcutaneous tumor weight. (F) Subcutaneous tumor formation rate at the indicated days. (G) CD26 and CD44 protein levels in subcutaneous tumors of HCT116‐miR‐372/373 and HCT116‐miR‐NC analyzed by western blotting. Error bars represent the SEM (*n* = 3). *P <* *0.05 by Student's *t*‐test.

To assess the self‐renewal ability, the stable cells were maintained in stem cell culture medium, which allows cells with the capacity of self‐renewal to form spheres. As expected, more spheres formed in miR‐372‐ and miR‐373‐expressing HCT116 cells (Fig. 2A) and competitive inhibition of miR‐372/373 reduced the sphere numbers in Caco‐2 cells (Fig. 2A). To examine drug resistance, HCT116 cells were treated with different doses of the chemotherapeutic drug 5‐fluorouracil for 72 h and cell viability was then determined. As expected, cells with enforced expression of miR‐372 and miR‐373 were more resistant to the 5‐fluorouracil treatment (Fig. 2B). In addition, the competitive inhibition of miR‐372/373 did not significantly reverse the effect of drug resistance in Caco‐2 cells, indicating that the efficiency of TuD may be not sufficiently high to reverse all of the effect of the overexpression of miRNA‐372‐373. A Transwell assay was performed to determine the migration and invasion potency of stable cells. Compared to the control group, enforced expression of miR‐372/373 enhanced the migration and invasion of HCT116 cells (Fig. 2C) and RKO cells (Fig. S2C). Taken together, these data suggested that miR‐372/373 promoted CSC properties of CRC cells.

Having obtained evidence indicating that miR‐372/373 are capable of impacting CRC phenotypes *in vitro*, we next investigated the role of miR‐372/373‐expressing cells *in vivo*. Stable cells were subcutaneously implanted into the dorsal flank of nude mice to form xenograft tumors. Mice bearing miR‐372/373‐expressing cells formed tumors much faster than the control group in the initial days after injection (Fig. 2D–F), indicating that ectopic expression of miR‐372/373 accelerates tumor formation of CRC *in vivo*. Western blot analysis showed that the protein levels of CSC markers CD44 and CD26 were upregulated in miR‐372/373‐overexpressing HCT116 cells in subcutaneously implanted tumors (Fig. 2G). These results indicate that overexpression of miR‐372/373 enhanced tumor formation and increased the stem cell population of CRC *in vivo*.

### 3.3 miR‐372/373 regulate stemness‐related signaling pathways by targeting differentiation genes

**Figure 3:**
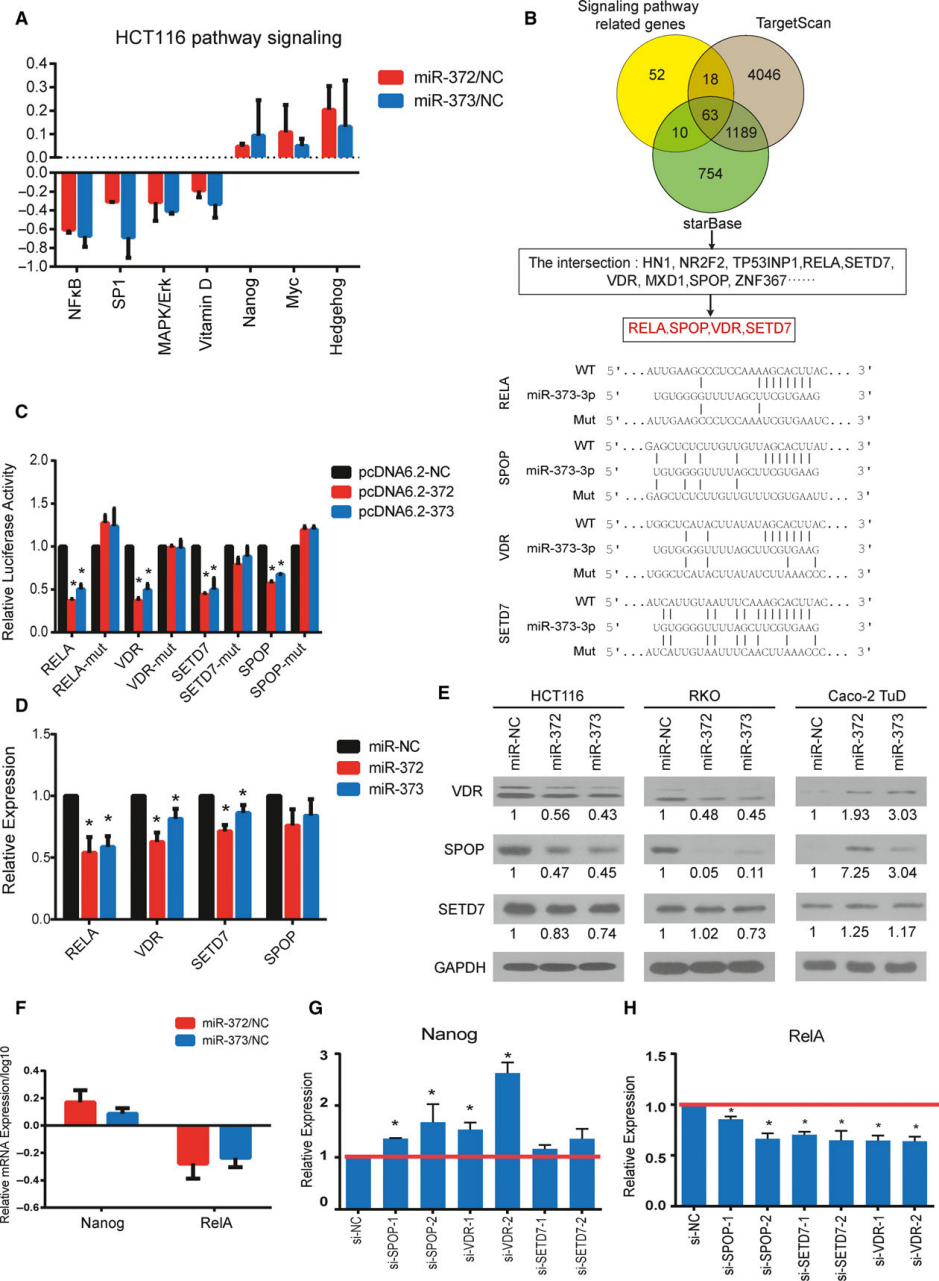
SPOP, VDR and SETD7 are direct targets of miR‐372/373 in CRC. (A) Relative activity of pathways suppressed and enhanced in miR‐372/373 transiently overexpressing cells compared to controls determined by luciferase reporter assays. (B) Venn diagrams showing the number of potential functional targets of miR‐372/373 predicted by targetscan and starbase and conserved and mutant binding sites of miR‐372/373 in the 3′ UTR of RELA, SPOP, VDR and SETD7. (C) 3′ UTR inhibition rate determined by luciferase reporter activity in HCT116 cells co‐transfected with pcDNA6.2‐miR‐372/373 and the wild‐type/mutant psiCheck2 3′ UTR luciferase reporter. (D) mRNA levels of RELA, SPOP, VDR and SETD7 in HCT116 miR‐372/373‐overexpressing cells determined by quantitative RT‐PCR. (E) Western blot analysis of expression of SPOP, VDR and SETD7 in the indicated cells. GAPDH served as the loading control. (F) mRNA of Nanog and RelA in HCT116 cells measured by quantitative RT‐PCR. (G, H) Quantitative RT‐PCR analysis of mRNA level of Nanog and RelA in HCT116 cells transfected with siRNAs for the indicated targets. Error bars represent the SEM (*n* = 3). *P <* *0.05 by Student's *t*‐test.

Differentiation or stemness‐related genes are generally involved in regulatory networks of key cell signaling pathways, and a single gene could be involved in different signaling pathways in different cellular contexts. To determine the major signaling pathways that are affected by miR‐372/373 in CRC cells, we profiled cell signaling pathways after both transiently and stably overexpressing miR‐372/373 in HCT116 cells. A Cignal 45‐pathway Reporter Array luciferase system was used to determine the activity of various core transcription factors and generate a signaling activity map. A group of pathways was significantly affected by miR‐372 and miR‐373 (Table S3). Particularly, we noted that stemness‐related pathways, such as Hedgehog, c‐Myc and Nanog signaling, were elevated and numerous pathways related to differentiation, such as NFκB, SP1, MAPK/Erk and vitamin D signaling, were markedly repressed when both transiently and stably overexpressing miR‐372/373 in CRC cells (Fig. 3A).

miRNAs exert their function by repressing target genes. To further clarify the underlying mechanism responsible for the signaling pathway changes caused by miR‐372/373, we predicted potential targets of miR‐372/373 using targetscan (Lewis *et al*., 2005) and starbase, version 2.0 (Li *et al*., 2014) and focused on the target genes that are involved in signaling pathway regulation (Fig. 3B and Table S4).

To validate the predicted targets that were suppressed by miR‐372/373 in CRC cells, we constructed luciferase reporter plasmids containing the 3′ UTR of each target. The 3′ UTR activity of RELA, VDR, SETD7, SPOP, TRERF1, ZNF367 and MTUS1 was significantly repressed by miR‐372/373 (Figs 3C and S3A). Although the miR‐372/373 target sites in the 3′ UTRs linked to the luciferase reporter were mutagenized, all mutant sites lost their response to miR‐372/373 (Figs 3C and S3A), indicating the site‐specificity of the repression. Quantitative RT‐PCR analysis revealed that miR‐372/373 downregulated the mRNA expression of these genes (Figs 3D and S3B), indicating a strong inhibitory effect. We then focused on and further investigated the protein levels of three new target genes, VDR, SETD7 and SPOP, all of which are involved in cell differentiation (Blomberg Jensen *et al*., 2012; Castano *et al*., 2016; Zhang *et al*., 2009; Zhou *et al*., 2017). Western blot analysis revealed that overexpression of miR‐372/373 inhibited the protein levels of all the targets in both HCT116 and RKO cells, with the exception of SETD7 in an assay with miR‐372 in RKO cells (Fig. 3E). Conversely, the protein levels of the three targets in Caco‐2 miR‐372/373‐TuD cells were elevated (Fig. 3E). These results indicated that the expression of SPOP, VDR and SETD7 was repressed by miR‐372/373 in CRC cells. It is worth noting that SPOP had a more significant change in response to the forced or alleviated expression of miR‐372/373.

We also confirmed the pathway profiling data in stable‐overexpressing cells by quantitative RT‐PCR. Consistently, mRNA levels of Nanog are enhanced by miR‐372/373 and mRNA of NFκB core factor RelA is suppressed by miR‐372/373 (Figs 3F and S3C). To determine whether disruption of SPOP, VDR and SETD7 contributes to the pathway change, we performed a transient knockdown of these genes using siRNAs in HCT116 cells. All siRNAs significantly suppressed the protein levels of their target genes (Fig. S4A,B) and, to a certain extent, this resulted in increased expression of the Nanog gene and suppressed expression of the RelA gene (Fig. 3G,H). These results indicated that miR‐372/373 target a series of genes to regulate signaling pathways in CRC cells.

### 3.4 Suppression of differentiation genes targeted by miR‐372/373 contributes to the phenotype of CSC

**Figure 4:**
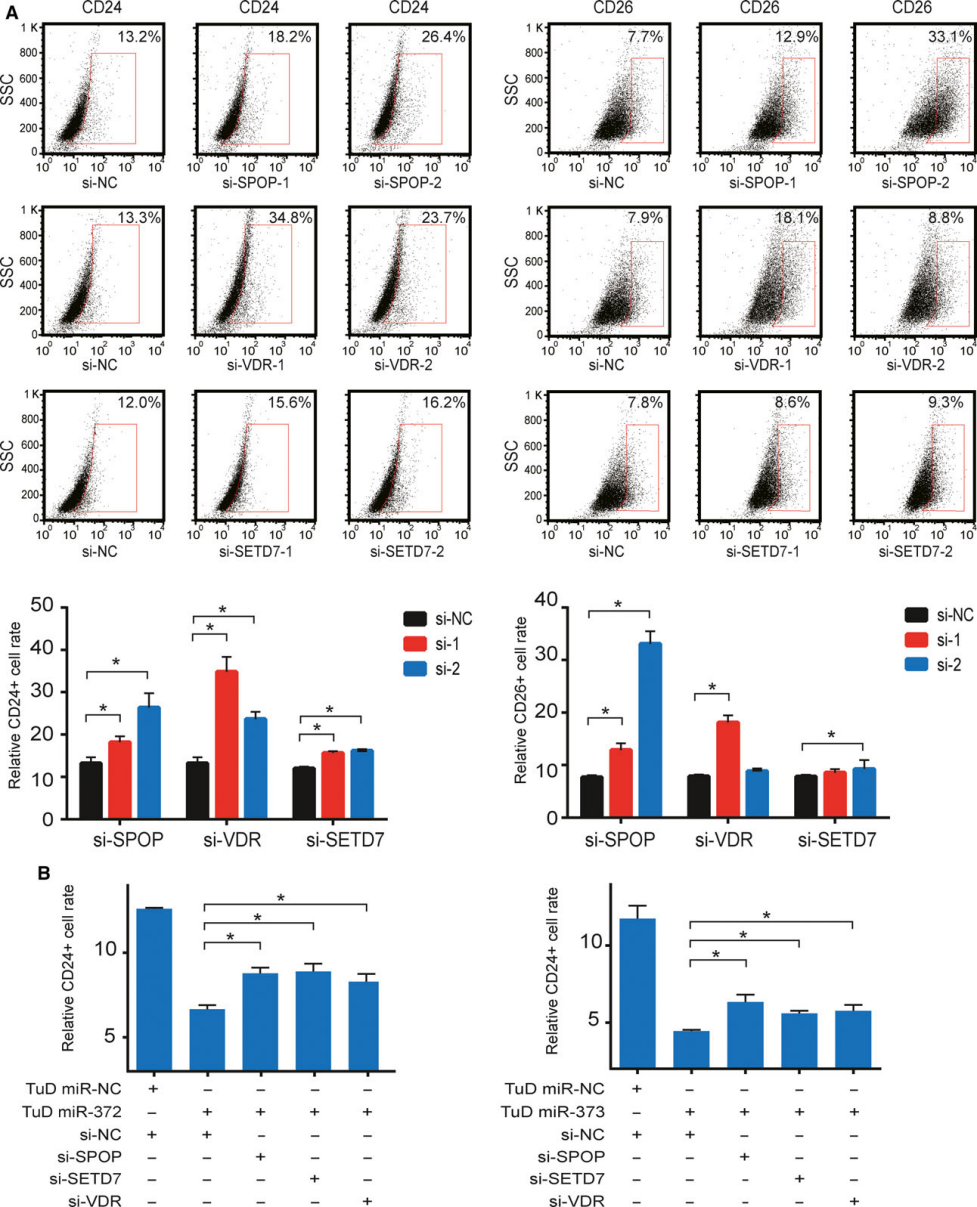
Knockdown of SPOP, VDR and SETD7 enhances the CRC stem cell phenotype. (A) Relative CD24+ and CD26+ cell populations in HCT116 cells transfected with the indicated siRNAs determined by FACS. (B) Relative CD24+ cell population measured by FACS in Caco‐2 miR‐372/373‐TuD cells transfected with the indicated siRNAs. Error bars represent the SEM (*n* = 3). *P <* *0.05 by Student's *t*‐test.

To determine the impact of SPOP, VDR and SETD7 on differentiation and stemness in colon cancer cell, we first tested the overexpression effects of these genes and found that SPOP and SETD7 positively regulated the expression of goblet cell differentiation marker HATH1, whereas VDR promoted the expression of columnar epithelial cell marker lactase in HCT116 cells (Fig. S5A,B). Next, we analyzed the proportion of CD24+ and CD26+ CSC populations by FACS and the protein levels of VDR, SETD7 and SPOP by western blotting in three CRC cell lines. The CD24+ and CD26+ CSC populations are high in Caco‐2 compared to RKO and HCT116 cells, whereas the expression of the target genes is low in Caco‐2 with the exception of VDR, which is not that much different (Fig. S5C,D). We further performed a transient knockdown of VDR, SETD7 and SPOP using siRNAs in HCT116 cells and analyzed the CD24+ and CD26+ CSC populations. As shown in Fig. 4A, siRNA silencing of SPOP, VDR and SETD7 significantly increased CD24+ and/or CD26+ cells, suggesting that CSC populations are expanded by suppressing these three target genes.

To further confirm that miR‐372/373 induced the CSC phenotype by targeting these genes in CRC, we knocked down each of the genes in Caco‐2 miR‐372/373‐TuD cells. Although inhibition of miR‐372 or miR‐373 attenuated the population of CD24+ cells in Caco‐2 cells, silencing all these targets by siRNAs at the same time relieved the suppression of the population of CD24+ cells in Caco‐2 miR‐372/373‐TuD cells (Fig. 4B). These results revealed that miR‐372/373 maintained the CSC phenotype, at least in part, by repressing SPOP, VDR and SETD7 in CRC.

**Figure 5:**
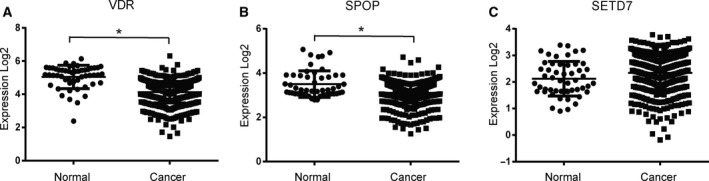
The expression of mRNA of (A) VDR, (B) SPOP and (C) SETD7 in CRC and adjacent normal colon samples from TCGA database. *P <* *0.05 by Student's *t*‐test.

In addition, we analyzed the expression of these genes in CRC patients from TCGA database. It is evident that the mRNA level of SPOP, VDR and SETD7 in CRC tissues is lower than in normal adjacent tissues (Fig. 5), indicating the tumor suppressive roles of these genes and the poor differentiation of CRC.

## 4 Discussion

It is assumed that 90% of CRC have an activation mutation of the Wnt/β‐catenin pathway (Schneikert and Behrens, 2007). In the present study, we showed that miRNA‐372/373 are upregulated in many CRC patient samples. This is consistent with the upregulated expression of Wnt/β‐catenin signaling because miR‐372/373 were transactivated by the pathway in CRC (Guo *et al*., 2016; Zhou *et al*., 2012). Furthermore, the expression level of miRNA‐372/373 may reflect different degrees of activities that correspond to the status of stemness or poor differentiation of CRC cells, as well as higher cancer progression and recurrence rates in patients (Table S5). Thus, it may prove to be novel diagnostic marker and therapeutic target for the clinical staging of CRC.

Multiple genes are determined to be new targets of miR‐372/373 in CRC cells in the present study. Interestingly, these genes are involved in diverse cellular biological processes with respect to differentiation. Among them, VDR is the nuclear receptor of 1,25‐dihydroxyvitamin D3, which has been reported to induce differentiation of testicular germ cells and T cells (Blomberg Jensen *et al*., 2012; Zhou *et al*., 2017). SETD7 is a lysine methyltransferase that mediates the methylation of proteins and is induced during the differentiation of human ESCs (Castano *et al*., 2016). SPOP, an E3 ubiquitin ligase adaptor, mediates the ubiquitination and degradation of Gli2 and inhibits Hedgehog signaling, which affects cell differentiation (Zhang *et al*., 2009). In the present study, these three genes were confirmed to positively regulate differentiation markers in CRC cells (Fig. S5). We have also silenced other potential miR‐372/373 targets, such as TRERF1, ZNF367 and MTUS1, by siRNA in HCT116 cells, and increased mRNA levels of the stem cell markers Nanog, Sox2, CD24, CD44 and CD26 were detected (Fig. S3D). TRERF1 and MTUS1 are reported to be involved in the differentiation in breast cancer cells (Gizard *et al*., 2006) and oral tongue squamous cell carcinoma (Ding *et al*., 2012), respectively. It has been reported that LAST2, DKK1 and TXNIP are targeted by miRNA‐372/373 in different cancer cells (Voorhoeve *et al*., 2006; Yan *et al*., 2011; Zhou *et al*., 2012). Interestingly, these genes are required for the differentiation of adipocytes (An *et al*., 2013), neurons (Mukhopadhyay *et al*., 2001) and natural killer cells (Gasiorek *et al*., 2015; Lee *et al*., 2005), respectively. From our analytic data, inhibiting the expression of differentiation genes to maintain stemness is the major function of miRNA‐372/373 in CRC and other cancers.

The cell signaling pathways regulated by miR‐372/373 in CRC cells were systematically demonstrated in the present study. miRNA‐372/373 significantly affected the activity of numerous pathways, indicating large‐scale changes in gene regulation networks. These results demonstrated that miRNAs are strong epigenetic regulators and a powerful driving force to transform cell status. Remarkably, among all of these pathways, NFκB is the one that is most repressed by miR‐372/373. It has been reported that the NFκB signal is able to trigger early differentiation of stem cells (Alvero *et al*., 2009; Luningschror *et al*., 2012; Nogueira *et al*., 2011; Pratt *et al*., 2009; Yang *et al*., 2010; Zhang *et al*., 2012). In both mouse and human ESCs, NFκB activity remains at a low level and increases remarkably during early differentiation (Luningschror *et al*., 2012; Yang *et al*., 2010; Zhang *et al*., 2012). Glioma initiation cells (GICs), comprising the only cell population with tumorigenic capacity in gliomas, are reported to have low NFκB activity that is upregulated during GIC differentiation (Nogueira *et al*., 2011). In addition, NFκB signaling is active in cancers and functions as a pro‐inflammatory pathway to boost the proliferation of cancer cells and cancer progenitor cells (Karin, 2006). Thus, the NFκB signaling pathway is tightly involved in the initiation and maintenance of cancer cell differentiation and the antagonism towards stemness in both ESCs and CSCs. By contrast, miR‐372/373 strongly suppress NFκB signaling to promote stemness, as clearly demonstrated in our pathway profiling analysis. The Wnt/β‐catenin pathway plays an important role in stem cells and has been reported to suppress NFκB in CRC (Deng *et al*., 2002). Consistently, in the present study, we have confirmed that activation of Wnt/β‐catenin signaling significantly inhibited the NFκB pathway in both HEK293T cells and HCT116 cells (Fig. S6). The results of the present study suggest that miR‐372/373, as key effectors of Wnt/β‐catenin signaling (Zhou *et al*., 2012), are undoubtedly major contributors to the crosstalk between the Wnt/β‐catenin and NFκB pathways in CRC cells.

CSCs comprise a subpopulation of tumor cells with stem cell properties and are considered to have the capacity for tumor initiation (Creighton *et al*., 2009; Li *et al*., 2008; Song and Miele, 2007). CSCs could survive and expand in immune‐deficient mice, differentiate to progeny cancer cells and fuel the growth of the tumor (Ricci‐Vitiani *et al*., 2007). However, when tumors are formed, the CSCs are relatively quiescent compared to their differentiated progeny (Chen *et al*., 2012). The origin of CSCs remains to be determined, especially with respect to whether CSCs are derived from normal stem cells or cancer cells. Recently, it was reported that breast tumor‐initiation cells originated in a region different from that of normal mammary stem cells, and it has been suggested that the apparently similar stem cell programs operating in tumor‐initiation cells and normal stem cells of corresponding normal tissues are likely to differ significantly (Ye *et al*., 2015). It is currently assumed that CSC populations are dynamic and that cancer stemness is not a rigid feature but can be modulated and even induced by many factors, such as the tumor microenvironment and genetic or epigenetic mutations (Jordan *et al*., 2006; Vermeulen *et al*., 2010). The results of the present study showed that miR‐372/373 impart a stem‐like phenotype to CRC cells by regulating multiple factors involved in differentiation and stemness. The dynamic transition from cancer cells to CSCs within the CRC population is delineated, indicating that cancer cells can be induced to acquire self‐renewal and chemotherapy resistance by stem cell‐specific miRNA regulation. From the results obtained in the present study, we suggest that stem cell‐specific RNAs such as miR‐372/373 are strong endogenous inducers of CRC stem cells. Intriguingly, overexpression of miR‐372/373 in the HCT116 cell line significantly increased other clusters of stem miRNAs, such as the miR‐302 family, by which it may amplify the stem cell‐like features in CRC cells (Xie *et al*., 2013). Our results are also consistent with studies of miR‐302, which was reported to be upregulated in CSCs in head and neck squamous cell carcinoma and prostate cancer (Bourguignon *et al*., 2012; Guo *et al*., 2017). These results provide evidence for cancer cell plasticity via miRNA‐driven epigenetic regulation, implying the origin of CSCs.

## 5 Conclusions

In summary, we report that excessive expression of the Wnt/β‐catenin signaling downstream effector miR‐372/373 enhances the stemness of CRC cells by targeting multiple genes/pathways involved in differentiation and stemness regulation. These findings highlight a crucial link between the stem‐specific miRNAs and the acquisition of CSC properties in the CRC, and open the possibility for future therapeutic intervention.

## Author contributions

L‐QW and PY performed the experiments and analyzed the data. L‐QW and L‐HQ conceived the study and wrote the paper. BL, Z‐RL, L‐SZ, Y‐HG and SL performed the experiments. L‐LZ, J‐HY, HX and HZ revised the manuscript.

## Supporting information


**Fig. S1.** Statistical correlation between miR‐372/373 and stem cell markers and efficiency of lentivirus generated stable miR‐372/373 ectopic or repressed cell lines.
**Fig. S2.** miR‐372/373 induced stem cell‐like phenotype of RKO cells and HCT116 cells and promoted migration and invasion potency in RKO cells.
**Fig. S3.** miR‐372/373 increased the expression of Nanog, suppressed the expression of RelA and directly targeted a series of targets to induce cancer stem cell phenotype.
**Fig. S4.** Knockdown efficiency of indicated siRNAs in HCT116 cells.
**Fig. S5.** The effect of enforced expression of VDR, SPOP and SETD7 on colon epithelial differentiation markers and the levels of VDR, SPOP, SETD7, CD24+ and CD26+ cells in colon cancer cell lines.
**Fig. S6.** RelA is suppressed by miR‐372/373 and Wnt signaling.
**Table S1.** Primers used for reverse transcription and real‐time PCR.
**Table S2.** Primers used for vector construction.
**Table S3.** Relative activity of pathways suppressed and enhanced in miR‐372/373 transiently and stably overexpressing cells determined by luciferase reporter assays.
**Table S4.** Predicted target genes involved in signaling pathway regulation.
**Table S5.** Clinico‐pathological variables and the expression of miR‐372 in colon cancer patients.
