# Supplementary material for: miR‐372 and miR‐373 enhance the stemness of colorectal cancer cells by repressing differentiation signaling pathways
Source: Mol Oncol. 2018 Sep 24;12(11):1949–64. doi: 10.1002/1878-0261.12376 (PMC6210048; doi:10.1002/1878-0261.12376)
Supplement: Supplementary file 1 — Fig. S1. Statistical correlation between miR‐372/373 and stem cell markers and efficiency of lentivirus generated stable miR‐372/373 ectopic or repressed cell lines. Fig. S2. miR‐372/373 induced stem cell‐like phenotype of RKO cells and HCT116 cells and promoted migration and invasion potency in RKO cells. Fig. S3. miR‐372/373 increased the expression of Nanog, suppressed the expression of RelA and directly targeted a series of targets to induce cancer stem cell phenotype. Fig. S4. Knockdown efficiency of indicated siRNAs in HCT116 cells. Fig. S5. The effect of enforced expression of VDR, SPOP and SETD7 on colon epithelial differentiation markers and the levels of VDR, SPOP, SETD7, CD24+ and CD26+ cells in colon cancer cell lines. Fig. S6. RelA is suppressed by miR‐372/373 and Wnt signaling. Table S1. Primers used for reverse transcription and real‐time PCR. Table S2. Primers used for vector construction. Table S3. Relative activity of pathways suppressed and enhanced in miR‐372/373 transiently and stably overexpressing cells determined by luciferase reporter assays. Table S4. Predicted target genes involved in signaling pathway regulation. Table S5. Clinico‐pathological variables and the expression of miR‐372 in colon cancer patients. [file MOL2-12-1949-s001.pdf]

Supporting information

Fig S1. Statistical correlation between miR-372/373 and stem cell markers and efficiency of lenti-virus generated stable miR-372/373 ectopic or repressed cell lines.

Fig S2. miR-372/373 induced stem cell-like phenotype of RKO cells and HCT116 cells and promoted migration and invasion potency in RKO cells.

Fig S3. miR-372/373 increased the expression of Nanog, suppressed the expression of RelA and directly targeted a series of targets to induce cancer stem cell phenotype.

Fig S4. Knockdown efficiency of indicated siRNAs in HCT116 cells.

Fig S5. The effect of enforced expression of VDR, SPOP and SETD7 on colon epithelial differentiation markers and the levels of VDR, SPOP, SETD7, CD24+ and CD26+ cells in colon cancer cell lines.

Fig S6. RelA is suppressed by miR-372/373 and Wnt signaling.

Table S1. Primers used for reverse transcription and real-time PCR.

Table S2. Primers used for vectors construction.

Table S3. Relative activity of pathways suppressed and enhanced in miR-372/373 transiently and stably overexpressing cells determined by luciferase reporter assays.

Table S4. Predicted target genes involved in signaling pathway regulation.

Table S5. Clinico-pathological variables and the expression of miR-372 in colon cancer patients.

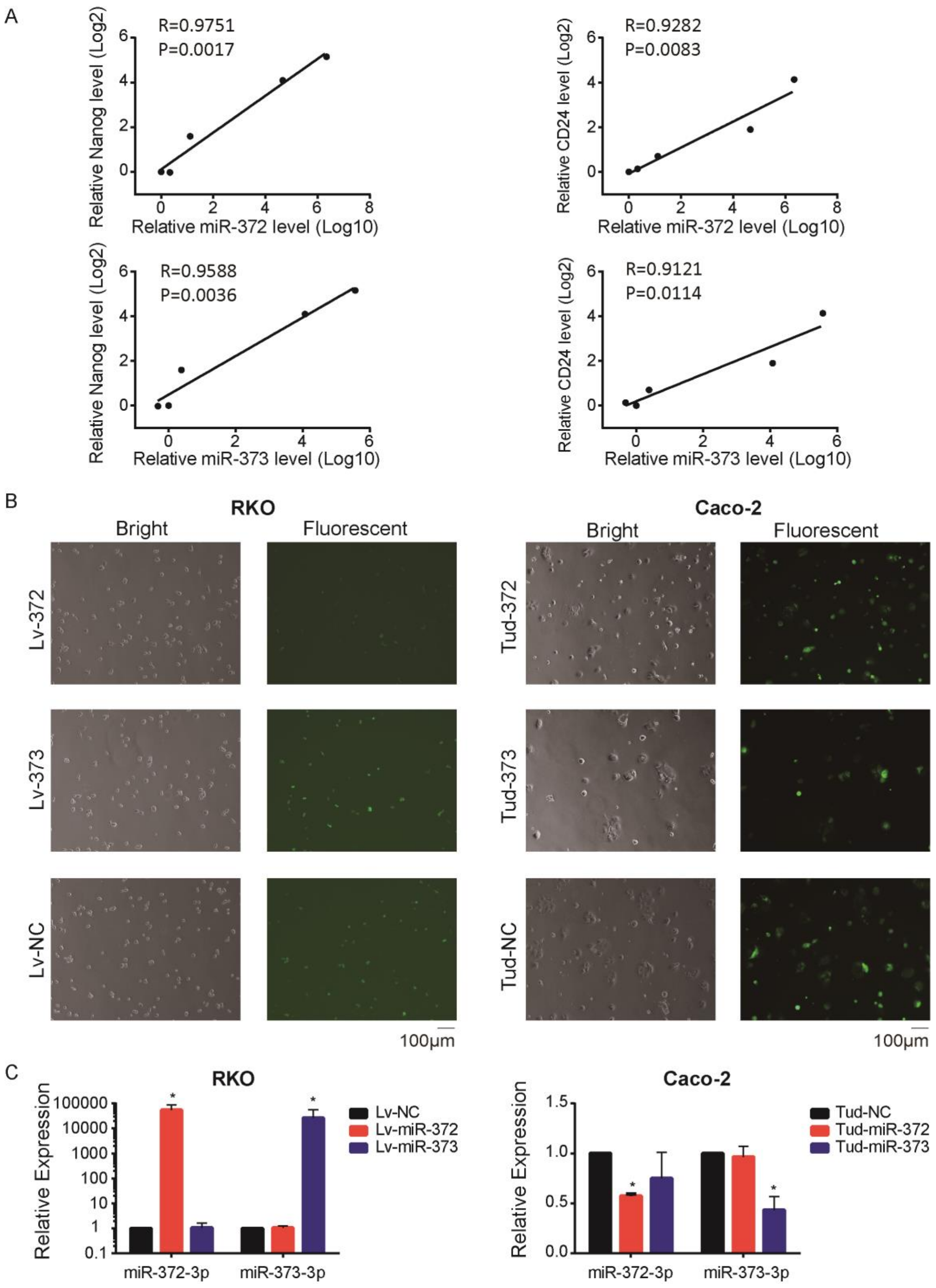

Figure S1. Statistical correlation between miR-372/373 and stem cell markers and efficiency of lenti-virus generated stable miR-372/373 ectopic or repressed cell lines. (A) Statistical correlation between miR-372/373 and Nanog/CD24 in colon cancer cell lines. (B) Representative images of GFP positive cell rate in RKO and Caco-2 stable cells. Scale bar: 100 μm. (C) Expression levels of miR-372 and miR-373 in indicated stable cells determined by qRT-PCR. The experiments were performed in triplicate. Error bars represent SEM. *p< 0.05 by Student's t-test.

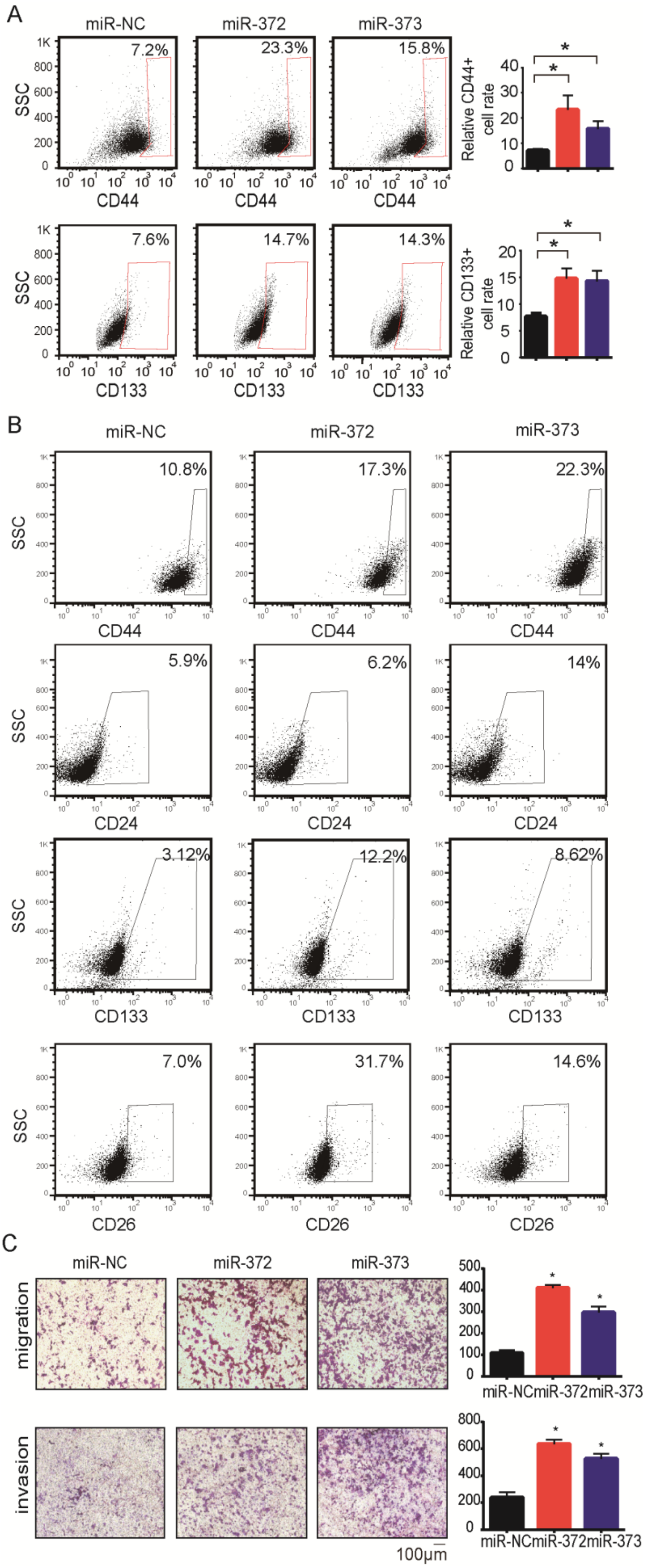

A
miR-NC
miR-372
miR-373
SSC
CD44
CD133
7.2%
23.3%
15.8%
7.6%
14.7%
14.3%
Relative CD44+ cell rate
Relative CD133+ cell rate
*
B
CD24
CD26
10.8%
17.3%
22.3%
5.9%
6.2%
14%
3.12%
12.2%
8.62%
7.0%
31.7%
14.6%
C
migration
invasion
100μm

Figure S2. miR-372/373 induced stem cell-like phenotype of RKO cells and HCT116 cells and promoted migration and invasion potency in RKO cells. (A) CD44 and CD133 in indicated HCT116 stable cells determined by FACS. (B) CD24, CD44, CD133 and CD26 levels in indicated RKO stable cells determined by FACS. (C) Representative images of migration and invasion assays in indicated RKO stable cells (left panel). The mean number of cells per visual field was determined in three randomly selected visual fields per chamber (right panel). Scale bar: 100 μm. The experiments were performed in triplicate. Error bars represent SEM. *$p < 0.05$ by Student's t-test.

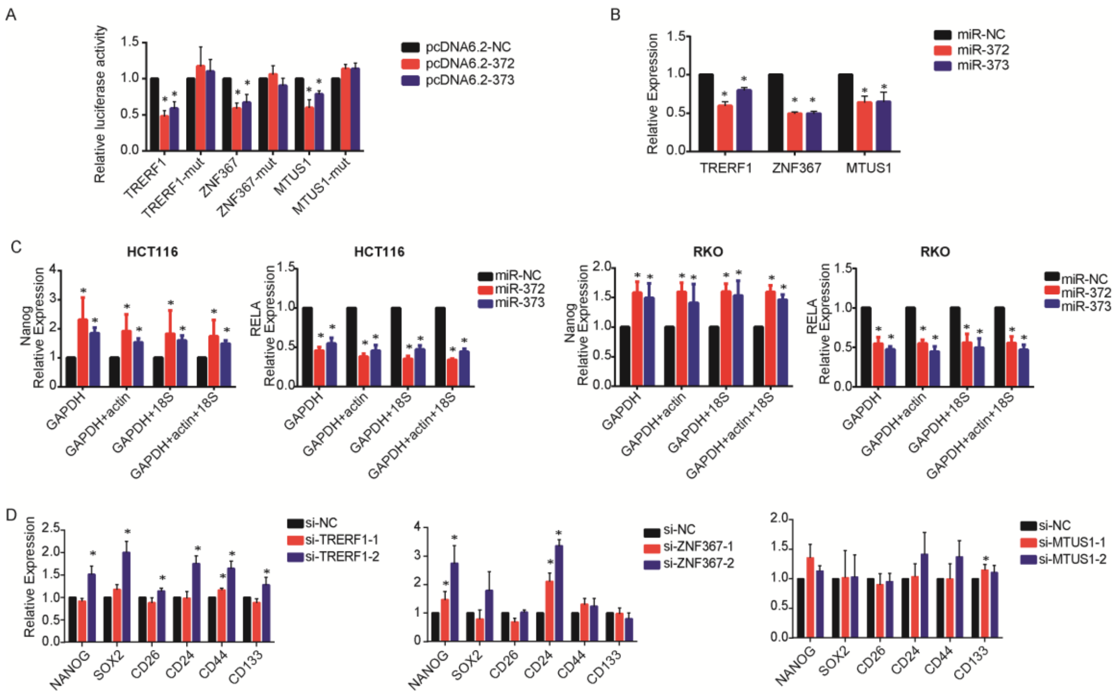

Figure S3. miR-372/373 increased the expression of Nanog, suppressed the expression of RelA and directly targeted a series of targets to induce cancer stem cell phenotype. (A) 3'UTR inhibition rate determined by luciferase reporter in HCT116 co-transfected with pcDNA6.2-miR-372/373 and wildtype/mutant psiCheck2 3'UTR luciferase reporters. (B) mRNA levels of TRERF1, ZNF367 and MTUS1 in HCT116 miR-372/373 overexpressed cells determined by qRT-PCR. (C) miR-372/373 increased the mRNA of Nanog and suppressed the mRNA of RelA in HCT116 and RKO cells. GAPDH and the geometric mean of two or three normalizers served as controls. (D) mRNA level of NANOG, SOX2, CD26, CD24,CD44 and CD133 in HCT116 cells transfected with indicated siRNAs determined by qRT-PCR. Error bars represent SEM, n=3. *p< 0.05 by Student's t-test.

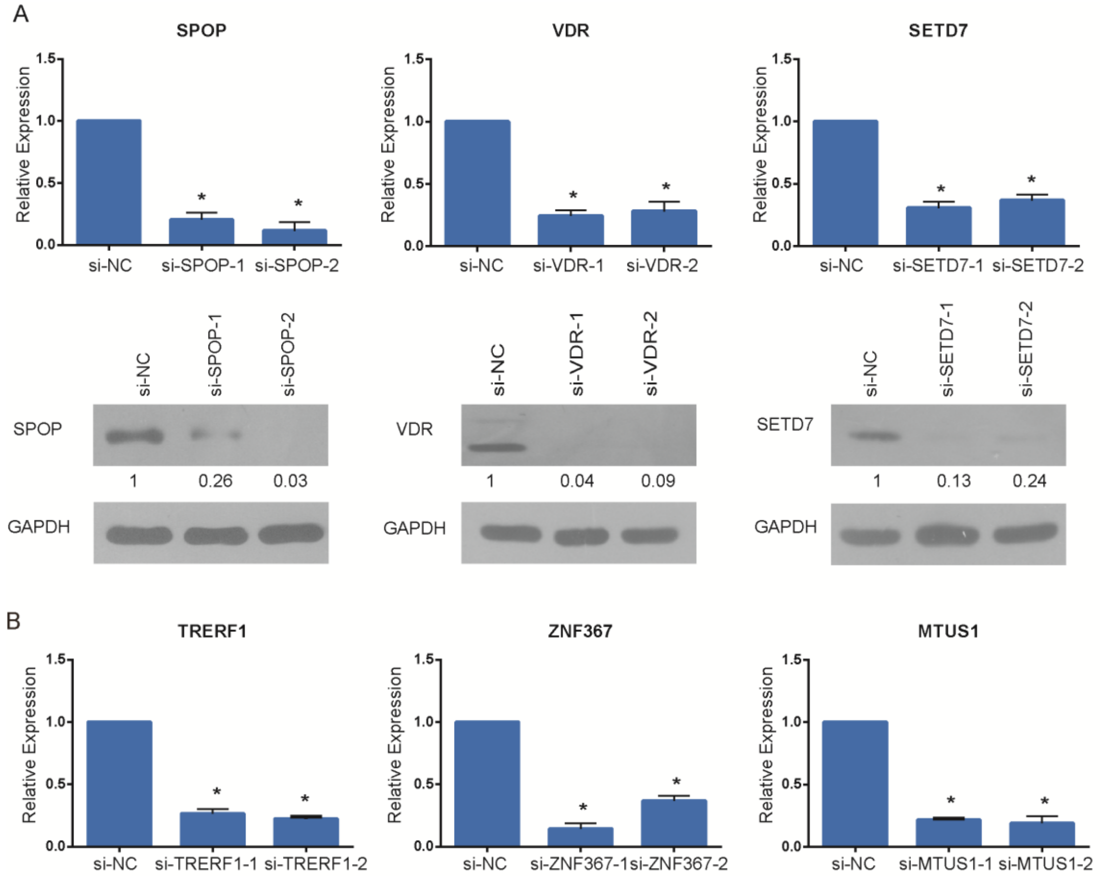

Figure S4. Knockdown efficiency of indicated siRNAs in HCT116 cells. (A) mRNA level and protein level of SPOP, VDR, SETD7 in HCT116 cells transfected with indicated siRNAs were determined by qRT-PCR and western blot. (B) mRNA level of TRERF1, ZNF367 and MTUS1 in HCT116 cells transfected with indicated siRNAs were determined by qRT-PCR. The experiments were performed in triplicate. Error bars represent SEM. *$p < 0.05$ by Student's t-test.

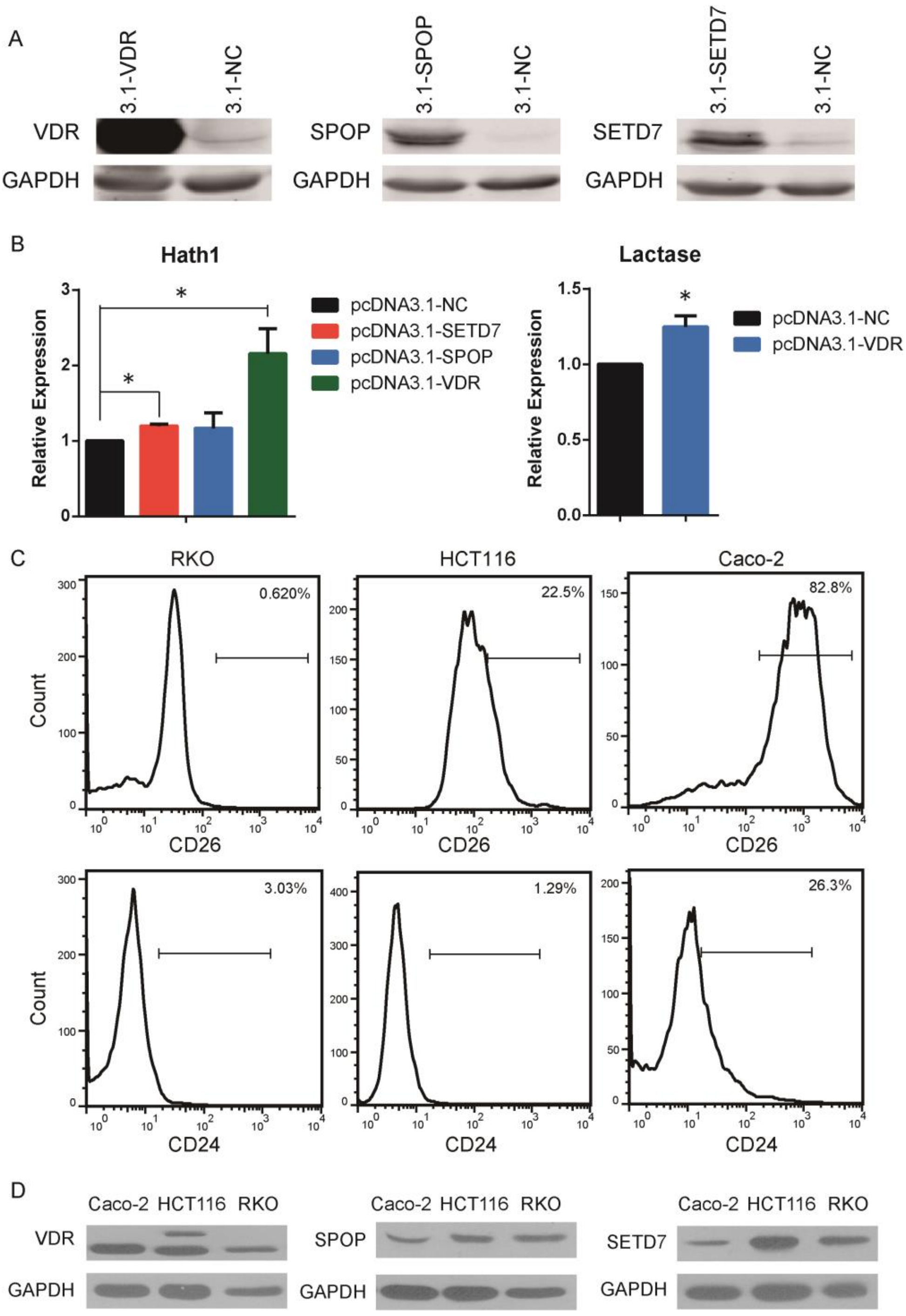

Figure S5. The effect of enforced expression of VDR, SPOP and SETD7 on colon epithelial differentiation markers and the levels of VDR, SPOP, SETD7, CD24+ and CD26+ cells in colon cancer cell lines. (A) Western blot analysis of expression of SPOP, VDR and SETD7 in the indicated pcDNA3.1 overexpressing HCT116 cells. (B) mRNA levels of Hath1 and Lactase in indicated HCT116 pcDNA3.1 overexpressing cells determined by qRT-PCR. (C) CD26+ and CD24+ cell populations in RKO, HCT116 and Caco-2 by FACS. (D) Protein levels of VDR, SPOP, SETD7 in RKO, HCT116 and Caco-2. Error bars represent SEM. *p< 0.05 by Student's t-test.

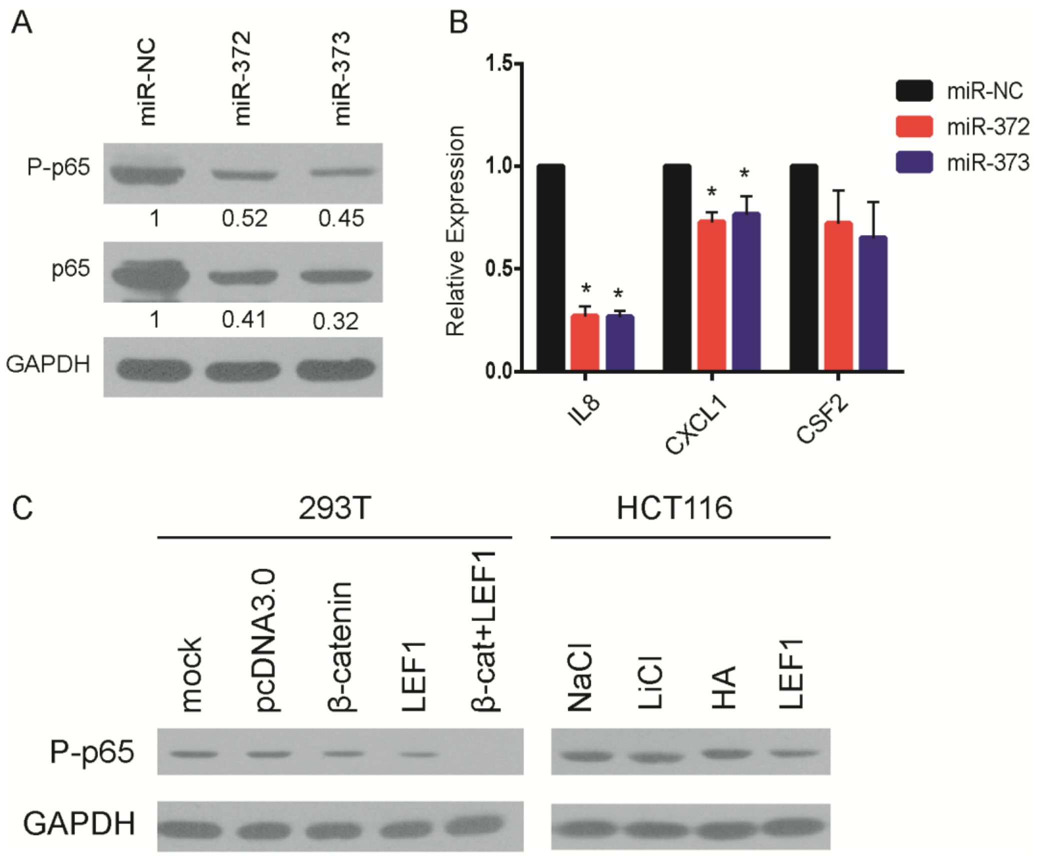

Figure S6. RelA is suppressed by miR-372/373 and Wnt signaling. (A) Protein level of RelA/p65 and p-p65 in HCT116 miR-372/373 overexpressed cells determined by qRT-PCR. (B) mRNA level of IL8, CXCL1 and CSF2 in HCT116 miR-372/373 overexpressed cells determined by qRT-PCR. (C) Western blot analysis of expression of p-p65 in the indicated cells. GAPDH served as the loading control. Error bars represent SEM, n=3. *p< 0.05 by Student's t-test.

Table S1: Primers used for reverse transcription and real-time PCR	
**Primer**	**Sequence(5'-3')**
**miRNA qRT-PCR primer**	
miR-372 RT	CTCAACTGGTGTCGTGGAGTCGGCAATTCAGTTGAGCACGCTCAA
miR-372 forward	TCGACAAAGTGCTGCGACATTT
miR-373-3p RT	CTCAACTGGTGTCGTGGAGTCGGCAATTCAGTTGAGCACACCCCA
miR-373-3p forward	TCGACGAAGTGCTTCGATTTTG
U6 RT	TTCACGAATTTGCGTGTCAT
U6 forward	CGCTTCGGCAGCACATATAC
U6 reverse	TTCACGAATTTGCGTGTCAT
SNORD48 RT	GGTCAGAGCGCTGCGGTGAT
SNORD48 foward	AGTGATGATGACCCCAGGTA
SNORD48 reverse	GGTCAGAGCGCTGCGGTGAT
5SrRNA RT	CCTACAGCACCCGGTATTCC
5SrRNA forward	TCTGATCTCGGAAGCTAAGCA
5SrRNA reverse	CCTACAGCACCCGGTATTCC
Universe reverse primer	CTCAACTGGTGTCGTGGAGTCGGC
**mRNA qRT-PCR primer**	
NANOG forward	ATGGAGGAAGGAAGAGGAGA
NANOG reverse	GATTTGTGGGCCTGAAGAAA
SOX2 forward	GCTTAGCCTCGTCGATGAAC
SOX2 reverse	AACCCCAAGATGCACAACTC
RELA forward	CGGGATGGCTTCTATGAGG
RELA reverse	CTCCAGGTCCCGCTTCTT
VDR forward	CATGTTGCGCTCCAATGAGTCC
VDR reverse	TCATGCAAGTTCAGCTTCTTCA
SETD7 forward	GGCAGTGTCCCCAGCCGCCATG
SETD7 reverse	GAGTAGGTGACTGTGCAGAACC
SPOP forward	AAACTCAGGCAGTGGATTTCAT
SPOP reverse	TAGGATTGCTTCAGGCGTTTGC
TRERF1 forward	AGAGAGTGAGGTGCCGAAGTCC
TRERF1 reverse	CATGGCCATTCAGTGCCTGTCG
ZNF367 forward	TTCATCCAGCAGAATCCGTTGT
ZNF367 reverse	CTGTCCACTTTGAACAAAGGCT
MTUS1 forward	CATCAACAGGACATCAAGTTAA
MTUS1 reverse	GCTGGAAACGCTTCAATTTGTC
IL8 forward	AGACAGCAGAGCACACAAGC

IL8 reverse	ATGGTTCCTTCCGGTGGT
CXCL1 forward	TCCTGCATCCCCCATAGTTA
CXCL1 reverse	CTTCAGGAACAGCCACCAGT
CSF2 forward	TCTCAGAAATGTTTGACCTCCA
CSF2 reverse	GCCCTTGAGCTTGGTGAG
OCT4 forward	GGTTCTCGATACTGGTTCGC
OCT4 reverse	GTGGAGGAAGCTGACAACAA
KLF4 forward	GTCAGTTCATCTGAGCGGG
KLF4 reverse	AGAGTTCCCATCTCAAGGCA
CD133 forward	ACTCCCATAAAGCTGGACCC
CD133 reverse	TCAATTTTGGATTCATATGCCTT
CD44 forward	CGGACACCATGGACAAGTTT
CD44 reverse	CGTGGAATACACCTGCAAAG
CD24 forward	ACAGCCAGTCTCTTCGTGGT
CD24 reverse	CCTGTTTTTCCTTGCCACAT
CD26 forward	CTGAACGCTCACTTCCGAG
CD26 reverse	CGACTGTCAGCTGTAGCATCA
HATH1 forward	TCCAGCAAACAGGTGAATGG
HATH1 reverse	TTGTAGCAGCTCGGACAAGG
Lactase forward	GTAGGAGGCTGGGAGAATGAGAC
Lactase reverse	CCCTGGTAAGCAATGACAAAGG
GAPDH forward	CCATGGGGAAGGTGAAGGTC
GAPDH reverse	GAAGGGGTCATTGATGGCAAC
Beta-actin forward	AGGCCAACCGCGAGAAGATGACC
Beta-actin reverse	GAAGTCCAGGGCGACGTAGCAC
18SrRNA forward	GTAACCCGTTGAACCCCATT
18SrRNA reverse	CCATCCAATCGGTAGTAGCG
**TUD sequence qRT-PCR primer**	
Tud-NC-F	GATCATCAACGATATCCCG
Tud-NC-R	TAGGATCATCTTGCGGTACG
Tud-372-F	GGATCATCAACACGCTCAAAT
Tud-372-R	GCTAGGATCATCTTGAAAGTG
Tud-373-F	AGGATCATCAACACACCCCAA
Tud-373-R	GCTAGGATCATCTTGGAAGTG

Table S2: Primers used for vectors construction	
**Primer**	**Sequences(5'-3')**
**Overexpression vectors**	
pcDNA6.2-miR-372 forward	CGCGGATCCAACCTGCGGAGAAGATACCA
pcDNA6.2-miR-372 reverse	CCGCTCGAGTCCTTTCACCATCTAACCCA
pcDNA6.2-miR-373 forward	CGCGGATCCCGACAGAGCAAGACTCATTC
pcDNA6.2-miR-373 reverse	CCGCTCGAGCTAAAATGGAGCATACAGAC
pLSNCG-miR-372 forward	CGCGGATCCAACCTGCGGAGAAGATACCA
pLSNCG-miR-372 reverse	CCGCTCGAGTCCTTTCACCATCTAACCCA
pLSNCG-miR-373 forward	CGCGGATCCCGACAGAGCAAGACTCATTC
pLSNCG-miR-373 reverse	CCGCTCGAGCTAAAATGGAGCATACAGAC
pcDNA3.1-VDR forward	CGCGGATCCGCCATGGAGGCAATGGCGGCCAG
pcDNA3.1-VDR reverse	CCGCTCGAGTCAGGAGATCTCATTGCCAA
pcDNA3.1-SPOP forward	CGCGGATCCGCCATGTCAAGGGTTCCAAGTCC
pcDNA3.1-SPOP reverse	CCGCTCGAGTTAGGATTGCTTCAGGCGTT
pcDNA3.1-SETD7 forward	CGCGGATCCGCCATGGATAGCGACGACGAGAT
pcDNA3.1-SETD7 reverse	CCGCTCGAGTCACTTTTGCTGGGTGGCCT
**Oligonucleotides used for cloning miR-372&373 targets motif to psiCHECK-2**	
RELA-WT forward	TCGAGCTGGGTTGCAGGGGATTGAAGCCCTCCAAAAGCACTTACGGATTCTGGTGGC
RELA-WT reverse	GCCACCAGAATCCGTAAGTGCTTTTGGAGGGCTTCAATCCCCTGCAACCCAGCTCGA
RELA-Mut forward	TCGAGCTGGGTTGCAGGGGATTGAAGCCCTCCAAATCGTGAATCGGATTCTGGTGGC
RELA-Mut reverse	GCCACCAGAATCCGATTCACGATTTGGAGGGCTTCAATCCCCTGCAACCCAGCTCGA

SPOP-WT forward	TCGAGTACAAGCATCAGAAGAGCTCTCTTGTTGTTAGCACTTATTGTTTGCAAGAACGC
SPOP-WT reverse	GGCCGCGTTCTTGCAAACAATAAGTGCTAACAACAAGAGAGCTCTTCTGATGCTTGTAC
SPOP-Mut forward	TCGAGTACAAGCATCAGAAGAGCTCTCTTGTTGTTTCGTGAATTTGTTTGCAAGAACGC
SPOP-Mut reverse	GGCCGCGTTCTTGCAAACAAATTCACGAAACAACAAGAGAGCTCTTCTGATGCTTGTAC
VDR-WT forward	TCGAGTATGGTAATAAAAATGGCTCATACTTATATAGCACTTACTTTGTTGCAAGTAGC
VDR-WT reverse	GGCCGCTACTTGCAACAAAGTAAGTGCTATATAAGTATGAGCCATTTTTATTACCATAC
VDR-Mut forward	TCGAGTATGGTAATAAAAATGGCTCATACTTATATCTTAAACCCTTTGTTGCAAGTAGC
VDR-Mut reverse	GGCCGCTACTTGCAACAAAGGGTTTAAGATATAAGTATGAGCCATTTTTATTACCATAC
SETD7-WT forward	TCGAGTGCTAAAACTCTTCATCATTGTAATTTCAAAGCACTTACCTGCTTCAAAACAGC
SETD7-WT reverse	GGCCGCTGTTTTGAAGCAGGTAAGTGCTTTGAAATTACAATGATGAAGAGTTTTAGCAC
SETD7-Mut forward	TCGAGTGCTAAAACTCTTCATCATTGTAATTTCAACTTAAACCCCTGCTTCAAAACAGC
SETD7-Mut reverse	GGCCGCTGTTTTGAAGCAGGGGTTTAAGTTGAAATTACAATGATGAAGAGTTTTAGCAC
TRERF1-WT forward	TCGAGCTTAAAGAATGGACTTCCTAGTACAATGTTGCACTTATTTTTTTTCTGAAGC
TRERF1-WT reverse	GGCCGCTTCAGAAAAAAAAATAAGTGCAACATTGTACTAGGAAGTCCATTCTTTAAGC
TRERF1-Mut forward	TCGAGCTTAAAGAATGGACTTCCTAGTACAATGTCTTAAACCTTTTTTTTCTGAAGC
TRERF1-Mut reverse	GGCCGCTTCAGAAAAAAAAAGGTTTAAGACATTGTACTAGGAAGTCCATTCTTTAAGC
ZNF367-WT forward	TCGAGTGTAATGTGGTATATAGAATTTTAATTTGGAGCACTTATAAGCTGGTAAGAGGC
ZNF367-WT reverse	GGCCGCCTCTTACCAGCTTATAAGTGCTCCAAATTAAAATTCTATATACCACATTACAC
ZNF367-Mut forward	TCGAGTGTAATGTGGTATATAGAATTTTAATTTGGTCGTGAATTAAGCTGGTAAGAGGC
ZNF367-Mut reverse	GGCCGCCTCTTACCAGCTTAATTCACGACCAAATTAAAATTCTATATACCACATTACAC
MTUS1-WT forward	TCGAGTTCGGCATCGACACGGACGTTGTTGCACAAAGCACTTAAAGAACGAGAGCATGC
MTUS1-W	GGCCGCATGCTCTCGTTCTTTAAGTGCTTTGTGCAACAACGTCCGTGTCG

T reverse	ATGCCGAAC
MTUS1-Mut forward	TCGAGTTCGGCATCGACACGGACGTTGTTGCACAATCGTGAATAAGAACGAGAGCATGC
MTUS1-Mut reverse	GGCCGCATGCTCTCGTTCTTATTCACGATTGTGCAACAACGTCCGTGTCGATGCCGAAC
**Oligonucleotides used for cloning Tud-miR-372&373 expression vectors**	
miR-372-tud forward	CGCGGATCCGACGGCGCTAGGATCATCAACACGCTCAAATGTCATCTGCAGCACTTTCAAGTATTCTGGTCACAGAATACAACACGCTCAAATGTCATCTGCAGCACTTTCAAGATGATCCTAGCGCCGTCTTTTTTGAATTCCGG
miR-373-tud forward	CGCGGATCCGACGGCGCTAGGATCATCAACACACCCCAAAATCATCTGAAGCACTTCCAAGTATTCTGGTCACAGAATACAACACACCCCAAAATCATCTGAAGCACTTCCAAGATGATCCTAGCGCCGTCTTTTTTGAATTCCGG
miR-NC forward	CGCGGATCCGACGGCGCTAGGATCATCAACGATATCCCGCCGCATCTGATCGTACCGCAAGTATTCTGGTCACAGAATACAACGATATCCCGCCGCATCTGATCGTACCGCAAGATGATCCTAGCGCCGTCTTTTTTGAATTCCGG

Table S3. Relative activity of pathways suppressed and enhanced in miR-372/373 transiently and stably overexpressing cells determined by luciferase reporter assays

	miR-372		miR-373	
Pathway	Stable	Transient	Stable	Transient
NFκB	0.85	0.30	0.75	0.27
SP1	0.63	0.48	0.83	0.21
MAPK/Erk	0.62	0.51	0.74	0.36
Retinoic Acid Receptor	0.78	0.84	0.79	0.80
KLF4	0.80	0.88	0.82	0.68
ER Stress	0.72	0.94	0.74	0.97
Oct4	0.82	0.88	0.85	0.96
C/EBP	0.96	0.85	0.88	0.79
Heavy Metal Stress	0.88	0.57	0.90	0.34
Type 1 Interferon	0.74	0.85	0.91	0.64
MEF2	0.88	0.99	0.92	0.89
Amino Acid Deprivation	0.79	0.71	0.93	0.81
cAMP/PKA	0.88	0.85	0.94	0.77
Vitamin D	0.92	0.87	0.94	0.79
Heat Shock Response	0.80	0.72	0.94	0.99
Liver X Receptor	0.79	0.93	0.95	0.76
EGR1	0.80	0.94	0.95	0.81
Androgen Receptor	0.84	0.79	0.97	0.93
Interferon Regulation	0.87	0.75	0.97	1.06
Negative Control	1.00	1.00	1.00	1.00
PPAR	1.09	0.90	1.01	1.00
p53	1.03	1.55	1.02	1.54
TGFβ	0.98	0.88	1.02	0.83
PI3K/Akt	0.99	1.03	1.03	1.05
STAT3	0.94	0.95	1.06	0.87
Interferon Gamma	1.27	0.99	1.13	1.00
Notch	1.47	0.96	1.06	0.90
Cell Cycle	0.91	0.94	1.16	1.16
Sox2	0.95	1.05	1.04	1.26
Myc	1.13	1.21	1.14	1.10
Nanog	1.28	1.11	1.19	1.27
Hedgehog	1.09	1.61	0.97	1.43

Table S4. Predicted target genes involved in signaling pathway regulation

Pathway	Pathway related target genes
NFκB	RELA,SETD7,RND3,TRIM8,PHLPP2,CADM1,TUSC2,TNFAP1,LAMP1
SP1	TRERF1,NAPEPLD,ARID4A,E2F7,TNFSF10,PTEN
MAPK	SIK1,RGMB,FOXO3,BCL2L11,MTUS1,TUSC3,BCL11B,ST13,FBXO31,TXNIP
Vitamin D	VDR,RORA,TP73
Sox2	RBL1,PFN2,PTEN
Nanog	TET1, LEFTY1,TRIM8,FAS,PTEN
Myc	HN1,BCL2L11,ARID4A,TRIM8,DMTF1,MXD1,PHLPP2,CTDSPL,RBL1,MDB2
Hedgehog	NR2F2, REST, PFN2,SPOP,ZNRF3,SIRT1

Table S5. Clinico-pathological variables and the expression of miR-372 in colon cancer patients

Characteristics[a]	Total	miR-372 positive		miR-372 negative		*P* value
		No.	%	No.	%	
Age (years)						0.073
<70	331	140	42.3	191	57.7	
≥70	257	90	35.0	167	65.0	
Gender						0.117
Female	278	118	42.4	160	57.6	
Male	310	112	36.1	198	63.9	
TNM stage						0.073
I	97	30	30.9	67	69.1	
II-IV	472	192	40.7	280	59.3	
Distant metastasis						0.911
Yes	87	33	37.9	54	62.1	
No	429	160	37.3	269	62.7	
Recurred/Progressed						0.023*
Yes	114	56	49.1	58	50.9	
No	397	148	37.3	249	62.7	
T stage						0.024*
I-II	114	34	29.8	80	70.2	
III-IV	472	195	41.3	277	58.7	

a. Clinical information are not available for all patients.

% means percentage within the row.

*means statistically significant ($P < 0.05$) by Chi square test.
